# Postoperative assessment of fracture reduction and osteosynthesis materials using photon-counting detector CT in maxillofacial trauma – a pilot study

**DOI:** 10.1007/s10006-025-01470-z

**Published:** 2025-10-11

**Authors:** Adib Al-Haj Husain, Victor Mergen, Sameena Sandhu, Maximilian Eberhard Hermann Wagner, Tristan T. Demmert, Hatem Alkadhi, Egon Burian, Thomas Flohr, Bernd Stadlinger, Peter Kessler, Suen An Nynke Lie, Harald Essig

**Affiliations:** 1 Department of Cranio-Maxillofacial and Oral Surgery, University Hospital Zurich, University of Zurich, Rämistrasse 100, Zurich, 8091 Switzerland; 2 Clinic of Cranio-Maxillofacial and Oral Surgery, Center of Dental Medicine, University of Zurich, Zurich, Switzerland; 3 Department of Cranio-Maxillofacial Surgery, GROW School for Oncology and Reproduction, Maastricht University Medical Centre, Maastricht, The Netherlands; 4 Diagnostic and Interventional Radiology, University Hospital Zurich, University of Zurich, Zurich, Switzerland; 5 Siemens Healthineers AG, Forchheim, Germany; 6 Department of Radiology and Nuclear Medicine, Maastricht University Medical Centre, Maastricht, the Netherlands

**Keywords:** Photon-counting detector computed tomography, Cone-beam computed tomography, Maxillofacial surgery, Fractures, Surgical procedure

## Abstract

**Purpose:**

To assess postoperative fracture reduction and visualization of titanium-based and bioresorbable osteosynthesis materials in maxillofacial trauma surgery using ultra-high-resolution photon-counting detector computed tomography (PCD-CT) compared with cone-beam computed tomography (CBCT).

**Methods:**

Fractures were induced in six cadaveric pig mandibles at the angle, body, and parasymphysis and were stabilized using various titanium and bioresorbable plate systems. Specimens were scanned with CBCT and dose-matched PCD-CT in the ultra-high resolution mode, applying standard and low-dose protocols. Two observers assessed fracture reduction and implant delineation using a 5-point visual analog scale. Fracture gap and osteosynthesis materials were quantitatively measured and compared to ground-truth values. Descriptive statistics and inter-reader agreement (weighted κ) were calculated.

**Results:**

PCD-CT enabled excellent assessment of fracture reduction and visualization of osteosynthesis materials, with perfect inter-observer agreement (Median = 5, IQR, 5–5; κ = 1.0, *p* < 0.001), whereas low-dose CBCT showed reduced image quality and lower reproducibility (Median = 4, IQR, 4–4; κ = 0.92; *p* < 0.001). Bioresorbable plates were not directly visible using either scanner, but the associated screw drill holes were reliably delineated. PCD-CT achieved the smallest measurement deviations of fracture gap and osteosynthesis materials compared with ground truth, and with superior reproducibility (κ = 0.74–0.84; ICC = 0.95–0.99; all *p* < 0.001), outperforming CBCT across most parameters.

**Conclusions:**

Ultra-high resolution PCD-CT combines superior visualization and precise measurements compared with CBCT even at low radiation dose. With further clinical validation, these findings highlight PCD-CT’s strong potential for perioperative imaging in maxillofacial trauma, particularly benefiting younger patients who require repeated scans.

## Introduction

In oral and maxillofacial trauma surgery, intraoperative imaging is primarily used to verify the positioning of osteosynthesis materials and guide necessary corrections. Postoperative imaging is performed to confirm fracture reduction and fixation, and in selected cases, is repeated after recovery of masticatory function to assess bone healing and long-term stability. Although early complications such as infections are usually diagnosed clinically, imaging provides an adjunct in inconclusive cases and is particularly valuable when postoperative findings raise suspicion of complications, e.g., material failure, that cannot be reliably assessed by clinical evaluation alone. Beyond this, imaging plays a crucial role in the long-term monitoring of complex reconstructions, serving as a basis for planning secondary surgical procedures [1]. Thus, image-guided perioperative clinical decision-making has further enhanced surgical precision and patient safety [2, 3].

Cone-beam computed tomography (CBCT) is widely regarded as the reference standard in perioperative dentomaxillofacial trauma imaging due to its high spatial resolution, comparatively low radiation dose, and broad clinical availability [4]. However, inherent limitations include considerable beam hardening artifacts from metal implants [5], as well as the cumulative radiation exposure [6], which is particularly relevant in young trauma patients who often require multiple scans throughout diagnosis, treatment, and follow-up.

Photon-counting detector computed tomography (PCD-CT) has recently been integrated into routine clinical practice and has emerged as a promising alternative to address these limitations [7]. Unlike conventional energy-integrating detectors, photon-counting detectors directly convert incident X-ray photons into an electrical signal. This enables the acquisition of scans with an ultra-high spatial resolution of less than 200 μm, which is comparable to that of conventional CBCT [8], with reduced electronic noise and enhanced radiation dose efficiency [9–11]. Ultra-high-resolution (UHR) images have proven successful in reducing artifacts from metal implants, which is particularly useful for visualizing surgical sites, where various metallic and bioresorbable osteosynthesis systems can compromise image quality and diagnostic interpretability of adjacent patho-anatomy. 

This ex vivo comparative study aimed to assess postoperative fracture fixation and visualization of titanium-based and bioresorbable osteosynthesis materials in maxillofacial trauma surgery using UHR PCD-CT compared with CBCT.

## Materials and methods

### Study design and ethics

Porcine mandibles were chosen due to their close anatomical and morphological similarity to the human craniofacial complex, as well as their well-established validation and widespread use as an experimental model in orofacial research [12]. The study protocol adhered to ethical standards as approved by the Office of Animal Welfare and 3R at the University of Zurich. Additionally, the reporting follows the ARRIVE (Animal Research: Reporting of In Vivo Experiments) guidelines.

### Specimen preparation

**Fig. 1:**
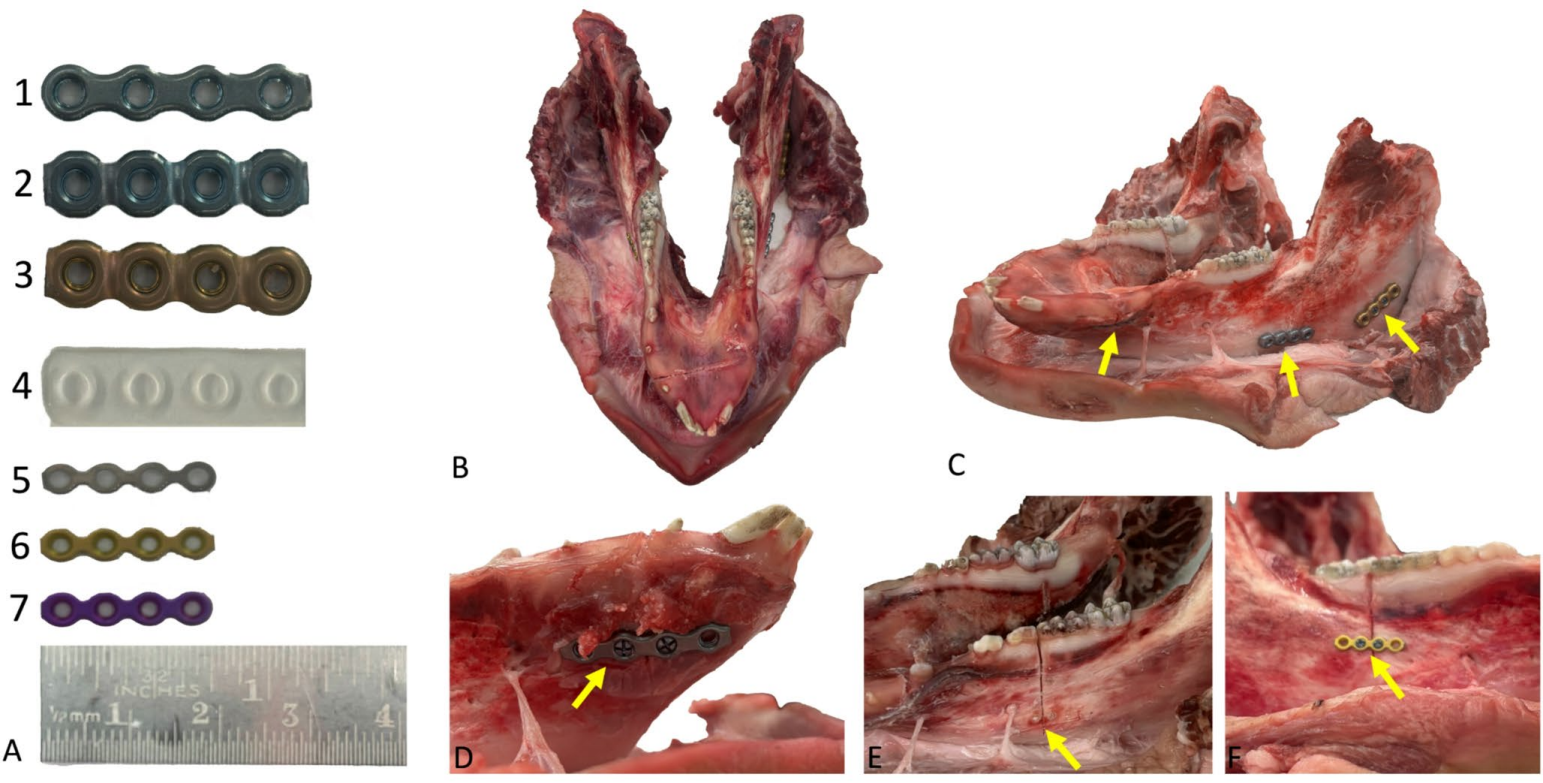
Specimen preparation. **A** Overview of the fixation systems: Titanium matrixMANDIBLE reconstruction plate (KLS Martin Group, Tuttlingen, Germany) in three thicknesses: (1) 2.0 mm (2), 2.5 mm, and (3) 2.8 mm; a bioresorbable Detla 1.7 copolymer plate (Stryker, Kalamazoo, MI, USA) with a thickness of 0.75 mm, and titanium microplates in thicknesses (KLS Martin Group, Tuttlingen, Germany) of (5) 0.6 mm (6), 0.8 mm, and (7) 1.0 mm. **B** Surgical exposure of the mandible following soft tissue dissection, with simulated fractures at the angle, body, and parasymphysis. **C** Subsequent fixation (indicated by arrows) at the three anatomical sites: angle, body, and parasymphysis. **D** Reconstruction of a parasymphyseal fracture using a titanium plate. **E** Application of a bioresorbable plate for fixation at the mandibular body. **F** Stabilization of the body region using a titanium microplate

The surgical procedures were carried out by M.E.H.W., a board-certified oral and maxillofacial surgeon with 17 years of practice; S.S., a board-certified oral and maxillofacial surgeon with 13 years of expertise; and A.A.H., a resident with 5 years of experience. After surgically dissecting the soft tissues, a total of 36 fractures were induced in a reproducible and controlled manner in six cadaveric pig mandibles across three anatomically vulnerable regions – the angle, body, and parasymphysis – resulting in 12 fractures per region. Each fracture was created using a saw with a standardized, uniform-thickness blade, with a new blade employed for every osteotomy to ensure consistent fracture patterns. To further standardize fracture morphology, all surgeons visually inspected and measured the fracture gap size, confirming comparability between mandibles. Fixation was then performed using pre-selected osteosynthesis plates, with the same number and positions of screws (two per plate) applied across all specimens to ensure consistency in the fixation method. The following fixation systems were used: titanium microplates with thicknesses of 0.6 mm, 0.8 mm, and 1.0 mm, respectively, anchored with maxDrive titanium screws (1.5 mm in diameter, lengths of 5 mm and 6 mm; all KLS Martin Group, Tuttlingen, Germany); a straight titanium matrixMANDIBLE reconstruction plate in thicknesses of 2.0 mm, 2.5 mm, and 2.8 mm, respectively, secured with corresponding titanium matrixMANDIBLE screws (diameter: 2.4 mm, 8 mm length; KLS Martin Group); and a bioresorbable copolymer Detla 1.7 plate (0.75 mm thickness) stabilized using 1.7 × 4 mm Delta screws (both Stryker, Kalamazoo, MI, USA). The osteosynthesis materials were assigned across all mandibles and fracture types using simple randomization, ensuring an equal frequency distribution of each plate at every anatomical site (Fig. 1).

### Image acquisition

#### CBCT data acquisition

All specimens were scanned using a CBCT system (NewTom 7G, QR Systems, Verona, Italy) applying the standard and low-dose scan settings recommended by the manufacturer. Due to the fixed y-coverage of the CBCT of 170 mm, which was less than the length of the mandible, two scans were performed per mandible in this experimental scenario. The first scan included the mandible angle but did not cover the menton. The second scan included the menton but did not cover the mandible angle. For the standard-dose protocol, scans were performed using a single X-ray source at 120 kV with automatic tube current selection and an exposure time per rotation of 3.8 s, resulting in a median dose length product (DLP) of 81.0 mGy ∗ cm (interquartile range (IQR), 79.7–82.8 mGy ∗ cm). Collimation width was 177 mm, and images were reconstructed with a field of view (FOV) of 170 × 170 mm^2^ and a matrix size of 440 × 440 pixels. For the low-dose scan protocol, the voltage was reduced to 100 kV with automatic tube current selection and an exposure time of 1.4 s, resulting in a median DLP of 15.4 mGy ∗ cm (IQR, 15.4–15.8 mGy ∗ cm). FOV and matrix size were constant.

#### PCD-CT data acquisition

The mandibles were scanned using a first-generation dual-source PCD-CT system (NAEOTOM Alpha; Siemens Healthineers AG, Forchheim, Germany), equipped with two cadmium telluride detectors. Scan acquisition was performed in the single-source, UHR mode employing a detector collimation of 120 × 0.2 mm. Tube voltage was set to 140 kV with tin pre-filtration and the pitch factor was 0.85. Both, the standard and low-dose scan protocols were dose-matched to the CBCT scans by manually adjusting the image quality level influencing the tube current modulation to match the DLP of the CBCT scans. For the standard dose protocol, the median DLP was 79.8 mGy ∗ cm (IQR, 79.0–81.6 mGy ∗ cm). For the low-dose protocol, the tube voltage and tin prefiltration remained unchanged. The median DLP was 14.3 mGy ∗ cm (IQR, 13.8–14.9 mGy ∗ cm).

Scans were reconstructed as UHR images with a slice thickness of 0.2 mm, an increment of 0.1 mm, using the sharp Hr76 kernel and quantum iterative reconstruction (QIR) at strength 4. FOV was 130 × 130 mm^2^, and the matrix size was 1024 × 1024 pixels.

### Image analysis

Qualitative and quantitative CBCT and PCD-CT image analyses were performed independently by two observers from the Department of Cranio-Maxillofacial and Oral Surgery: Observer A (S.S.) and Observer B (A.A.H.), with 13 and 4 years of experience, respectively, in perioperative dentomaxillofacial imaging.

All analyses were performed using the local Picture Archiving and Communication System (PACS) (DeepUnity Diagnost, release v.1.1.1.2, Dedalus HealthCare, Bonn, Germany), in the same controlled lighting environment. Reviewers were allowed to adjust window levels and magnification settings according to their personal preferences. For CBCT scans, the two acquisitions per mandible were not merged, and each scan was evaluated independently. Thus, observers were blinded to each other’s assessments and the imaging protocol, and the order of image evaluation was randomized with all identifiers removed to minimize potential bias. Prior to the evaluation, a calibration session was conducted to standardize the assessment process. During this session, three randomly selected cases, along with their respective imaging modalities and dose protocols, were reviewed with the guidance of one of the study’s principal investigators (A.A.H.) to ensure consistency and reduce potential assessment ambiguities or bias.

### Qualitative assessment

Postoperative delineation of fracture reduction, osteosynthesis material positioning, and structural contours of osteosynthesis plates and screws, including heads, drives, and threads were evaluated using a modified 5-point analog visual scale.

Delineation of fracture reduction and osteosynthesis material positioning were graded applying the following modified visual analog scale [13]: 5, excellent visualization: fracture reduction and osteosynthesis material positioning are clearly visible, 4, good visualization: minor limitations without relevant impact on postoperative assessment of fracture reduction or osteosynthesis material positioning; 3, adequate visualization: noticeable limitations with clear impact on the postoperative assessment of fracture reduction and osteosynthesis material positioning; 2, poor visualization: postoperative assessment of fracture reduction and osteosynthesis material positioning is severly limited; 1, non-assessable visualization: postoperative assessment of fracture reduction and osteosynthesis material positioning is not possible.

Visualization of structural contours of osteosynthesis plates and screws were separately assessed applying the following modified visual analog scale was applied: 5, excellent visualization: complete and sharp visualization of the entire plate contours and screw components (including heads, drives, and threads) without any loss of detail; 4, good visualization: clear visualization of plate contours and screw components with only minimal reduction in sharpness and definition; 3, adequate visualization: visualization of plate contours and screw threads is generally possible, with moderate reduction in sharpness and definition, 2, poor visualization: plate contours and screw threads are only partially visible with severe reduction in sharpness and definition; 1, non-assessable visualization: plate contours and screw threads are indistinct and cannot be reliably assessed.

### Quantitative assessment

The same two observers (A.A.H. and S.S.) independently performed quantitative measurements in defined axial, coronal, and/or sagittal plane reconstructions across all imaging protocols. Parameters assessed included the maximum extent of the fracture gap, osteosynthesis plate thickness and length, and screw length and diameter, each measured at their maximum dimension in millimeters. The obtained measurements were subsequently compared to the ground-truth dimensions. The ground-truth fracture gap size was measured in all specimens using a digital caliper, while osteosynthesis material dimensions were measured only when they had been modified and thus deviated from the manufacturer’s original specifications.

### Statistical analysis

Statistical analyses were conducted using IBM SPSS Statistics software (version 29.0.2.0, IBM Chicago, IL, USA). Descriptive statistics were used to analyze the collected data, calculating appropriate measures, including medians, interquartile ranges (IQR), and minimum and maximum values as needed. Inter-reader reliability was assessed using weighted kappa statistics, with the strength of agreement categorized as follows: poor (< 0), slight (0-0.2), fair (0.21–0.4), moderate (0.41–0.6), substantial (0.61–0.8), and almost perfect (0.81-1) [14]. Additionally, Intraclass Correlation Coefficient (ICC) was assessed for continuous measurements and interpreted as follows: poor (< 0.5), moderate (0.5–0.75), good (0.75–0.9), and excellent (>0.9). Differences in quantitative assessment were expressed in terms of absolute and relative differences. A two-sided significance level of α = 0.05 was applied for all statistical tests.

## Results

Two blinded observers independently evaluated a total of 24 image datasets, comprising six mandibles, each imaged using CBCT and dose-matched PCD-CT at both standard- and low-dose levels.

### Qualitative results

**Fig. 2:**
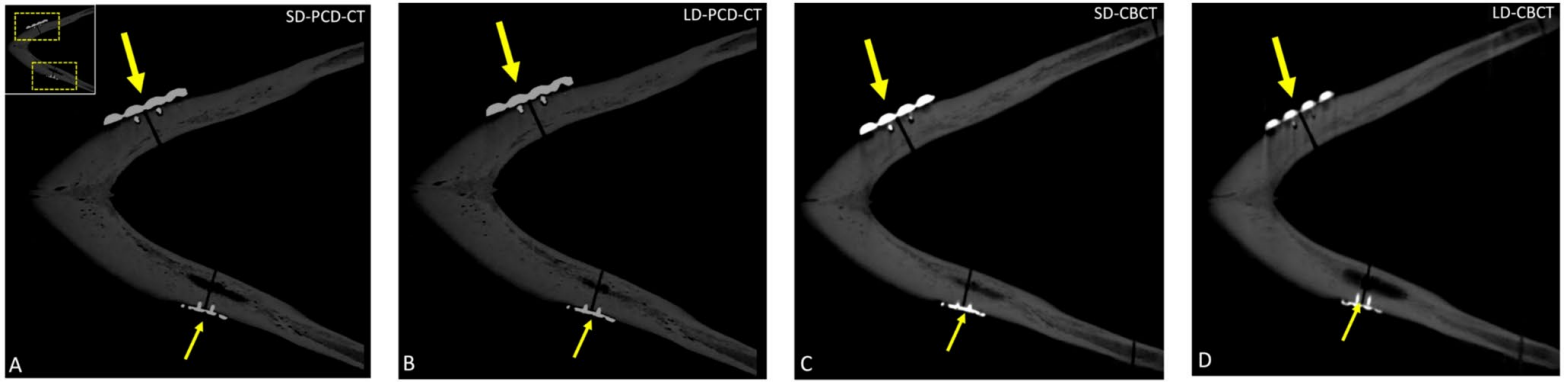
Visualization of bilateral mandibular fracture reduction and osteosynthesis material positioning. On one side, a 2.8 mm titanium reconstruction plate is secured with matrixMANDIBLE screws (diameter: 2.4 mm, length: 8 mm) (long thick arrow). On the contralateral side, a 0.8 mm titanium microplate is anchored with two maxDrive screws (diameter: 1.5 mm, length: 5 mm) (short thin arrow) (all KLS Martin Group, Tuttlingen, Germany). Imaging was performed using **A** standard and **B** low-dose photon-counting computed tomography (PCD-CT) and with dose-equivalent standard-dose **C** and low-dose **D** cone-beam computed tomography (CBCT)

Postoperative delineation of fracture reduction and osteosynthesis material positioning was rated as excellent on both standard- and low-dose UHR PCD-CT, as well as on standard-dose CBCT, with all observers assigning a median score of 5 (IQR: 5–5) and demonstrating perfect inter-observer agreement (κ = 1.0; 95% CI: 1.0–1.0; *p* < 0.001). In contrast, low-dose CBCT received lower ratings (median score: 4; IQR: 4–4) and inter-observer agreement (κ = 0.92; 95% CI: 0.77–1.0; *p* < 0.001), indicating superior performance of UHR PCD-CT at reduced dose levels (Fig. 2).

**Fig. 3:**
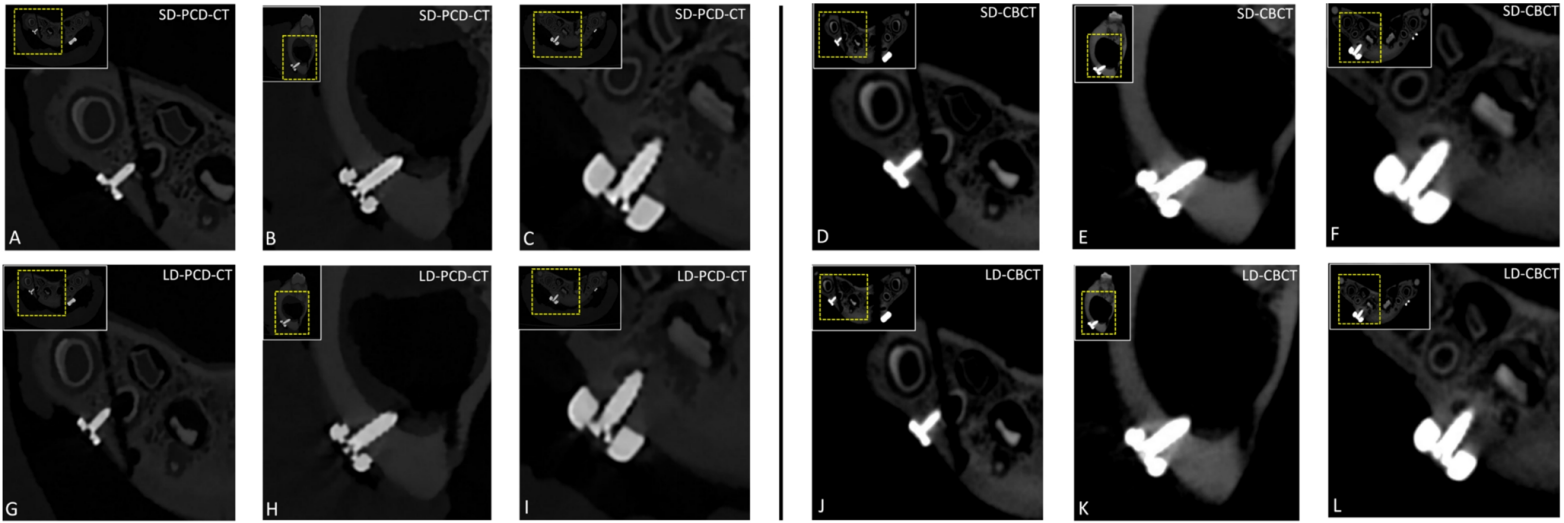
Mandibular fracture fixations using titanium-based microplates and reconstruction plates of varying thicknesses, as well as screws of different lengths and diameters, shown in standard-dose photon-counting computed tomography (PCD-CT) **A–C**, dose-equivalent cone-beam computed tomography (CBCT) **D–F**, low-dose PCD-CT **G–I**, and corresponding dose-matched low-dose CBCT **J–L**

A similar trend was observed in the visualization of structural contours of osteosynthesis plates and screws. Both standard-dose and low-dose UHR PCD-CT enabled complete and sharp visualization of the entire plate contours without any loss of detail (median score: 5, IQR: 5–5; for both κ = 1.0). UHR PCD-CT outperformed dose-matched CBCT scans for the delineation of screw heads, drive recesses, and threads at both standard and low doses. Inter-observer agreement was only perfect for the standard-dose UHR PCD-CT (κ = 1.0; 95% CI: 1.0–1.0; *p* < 0.001) and was lower for the CBCT scans (κ = 0.75–0.84; all *p* < 0.001). The lowest ratings were observed for low-dose CBCT in the delineation of both osteosynthesis plates and screws (median score: 3 or 4; with IQR: 3–3 or 3–4; κ = 0.59–0.84), where visualization of plate contours and screw threads remained generally possible, but showed moderate reduction in sharpness and definition (Fig. 3).

**Fig. 4:**
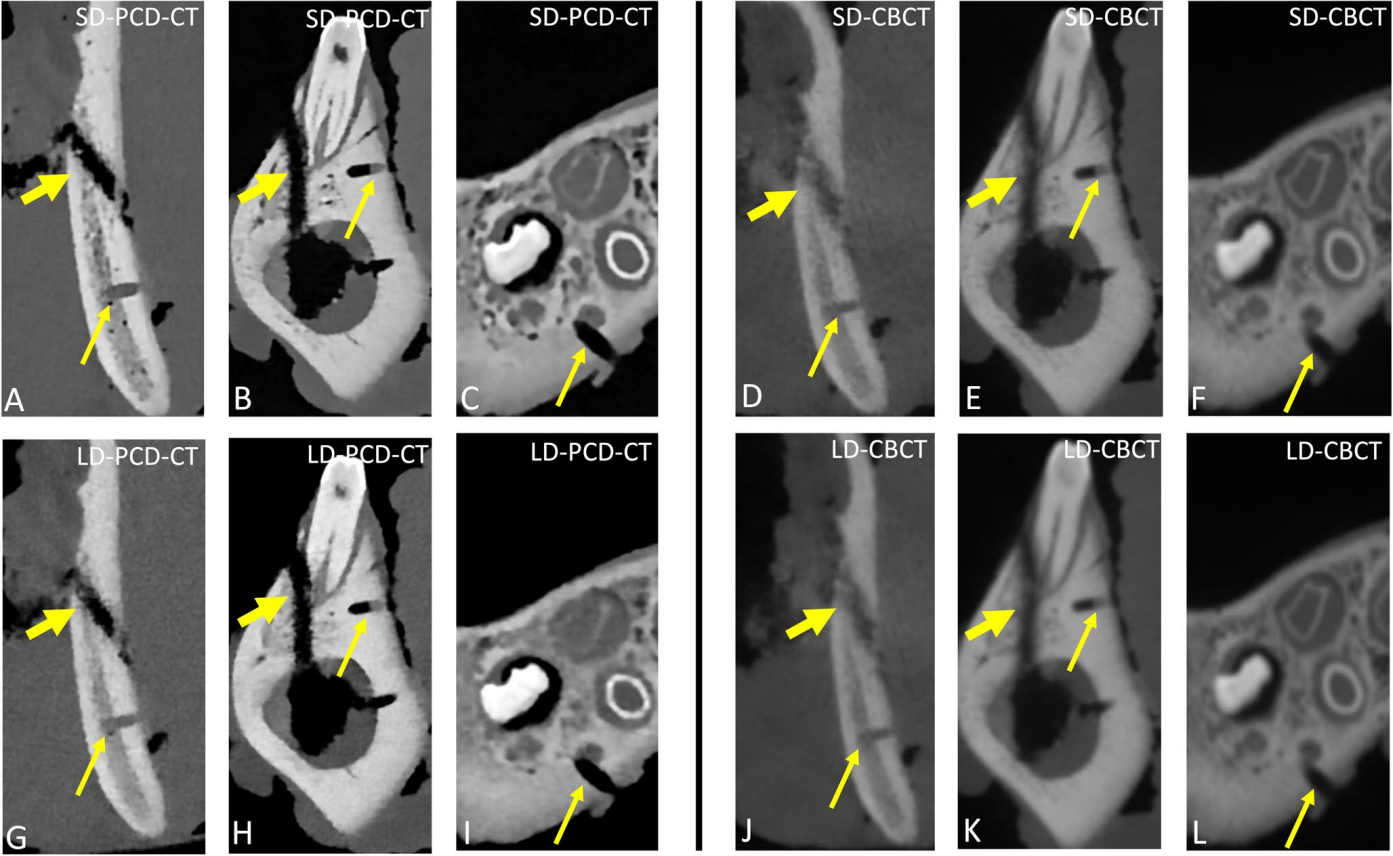
Coronal images illustrating mandibular angle **A**,** D**,** G**,** J**, body **B**,** E**,** H**,** K**, and parasymphyseal **C**,** F**,** I**,** L** fractures, each stabilized using a bioresorbable Delta 1.7 copolymer plate (0.75 mm thickness) and 1.7 × 4 mm Delta screws (both from Stryker, Kalamazoo, MI, USA), as visualized across all imaging modalities and their corresponding dose-matched protocols

The use of bioresorbable copolymer plates enabled an excellent assessment of fracture reduction, regardless of the chosen imaging modality or protocol. However, the osteosynthesis material itself was not directly visible, with only the screw drill holes being delineable (Fig. 4).

**Fig. 5:**
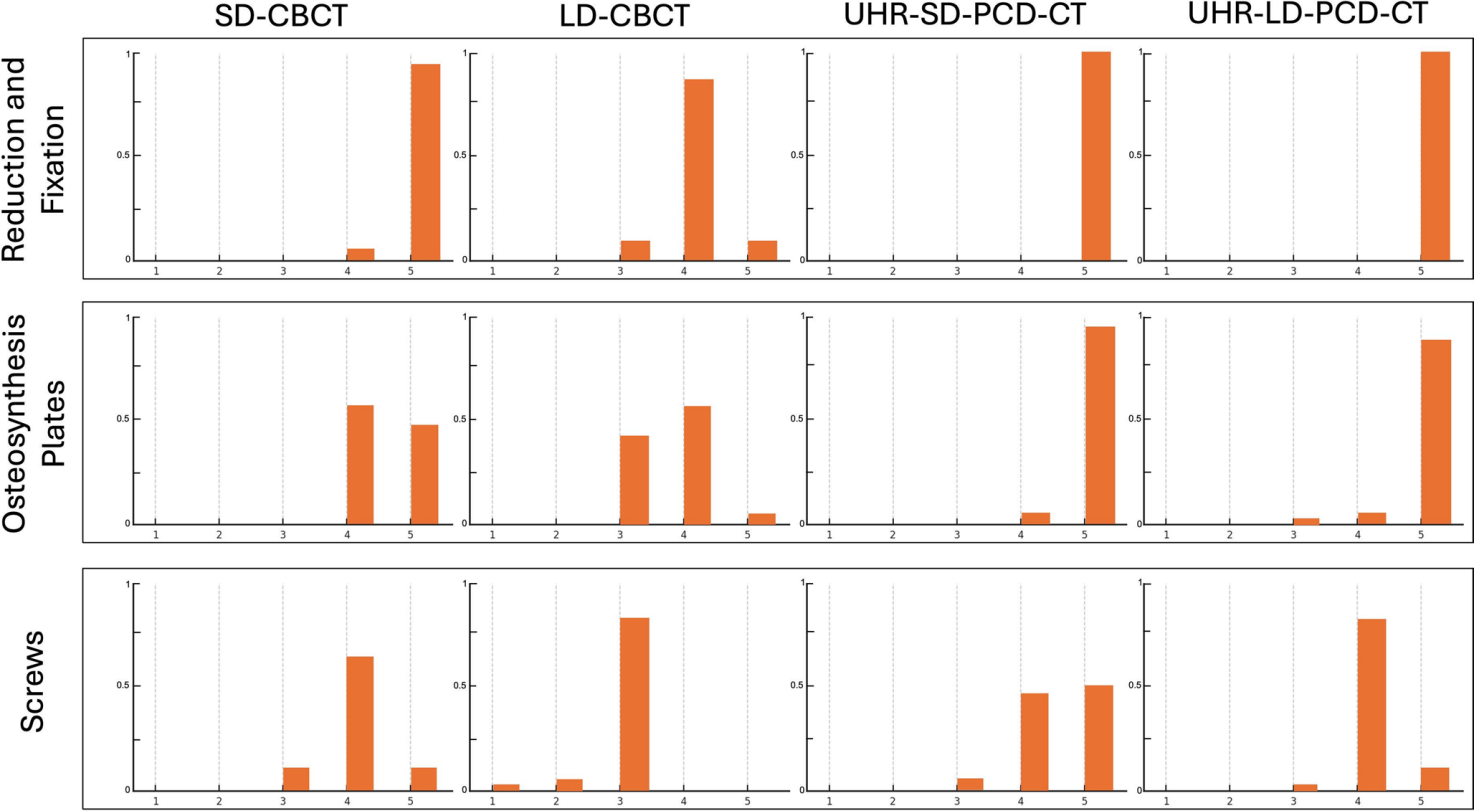
Distribution of ordinal visual grading scores from qualitative assessments, evaluating fracture reduction and osteosynthesis material positioning, as well as visualization of structural contours of osteosynthesis plates and screws. Ratings are shown for each imaging modality using corresponding dose-matched protocols (scale: 5 = best visualization, 1 = poorest)

Results are summarized in Table 1 as medians with interquartile ranges, alongside corresponding inter-observer agreement. The frequency distribution of visual grading scores is presented in Fig. 5.

**Table 1:** Qualitative assessment of parameters relevant to postoperative delineation of fracture reductionand osteosynthesis materials positioning, including visualization of structural contours of plates and screws, specifically the screw heads, drive recesses, and threads, was performed using a cone-beam CT (CBCT) and a dose-matched photon-counting detector CT (PCD-CT). A modified 5-point ordinal visual scale was used (5 = excellent, 1 = extremely poor). Two independent observers (A = attending, B = resident) performed the evaluations. Results are presented as medians with interquartile ranges in parentheses. Inter-observer agreement was assessed using weighted statistics (κ), where κ = 1 indicates perfect reliability and κ = 0 indicates no reliability beyond chance

	Imaging protocol	Observer A	Observer B	Inter-observer agreement (κ) and 95% confidence interval (CI)
Fracture reduction and osteosynthesis materials positioning	SD-CBCT LD-CBCT UHR-SD-PCD-CT UHR-LD-PCD-CT	5 (5–5) 4 (4–4) 5 (5–5) 5 (5–5)	5 (5–5) 4 (4–4) 5 (5–5) 5 (5–5)	1.0 (1.0–1.0); *p* < 0.001 0.92 (0.77-1.0); *p* < 0.001 1.0 (1.0–1.0); *p* < 0.001 1.0 (1.0–1.0); *p* < 0.001
Osteosynthesis plates	SD-CBCT LD-CBCT UHR-SD-PCD-CT UHR-LD-PCD-CT	4 (4–5) 3 (3–4) 5 (5–5) 5 (5–5)	4 (4–5) 4 (3–4) 5 (5–5) 5 (5–5)	0.79 (0.56-1.00); *p* < 0.001 0.59 (0.36–0.82); *p* < 0.001 1.0 (1.0–1.0); *p* < 0.001 1.0 (1.0–1.0); *p* < 0.001
Screws	SD-CBCT LD-CBCT UHR-SD-PCD-CT UHR-LD-PCD-CT	4 (4–4.25) 3 (3–3) 4.5 (4–5) 4 (4–4)	4 (4–4) 3 (3–3) 4.5 (4–5) 4 (4–4)	0.75 (0.58–0.98); *p* < 0.001 0.84 (0.63-1.0); *p* < 0.001 1.0 (1.0–1.0); *p* < 0.001 0.89 (0.69-1.0); *p* < 0.001

*CBCT* Cone-Beam Computed Tomography, *PCD-CT* Photon-Counting Detector Computed Tomography, *UHR* Ultra-High Resolution, *SD* Standard-Dose, *LD* Low-Dose

### Quantitative results

Regarding the quantitative assessments of absolute and relative differences (in millimeters and percentages) between radiological measurements and ground truth, UHR PCD-CT at both standard and low-dose protocols consistently demonstrated the smallest deviations from the ground truth and the highest interobserver agreement (κ = 0.74–0.84, ICC = 0.95–0.99; all *p* < 0.001). In contrast, CBCT showed greater measurement variability, particularly in low-dose protocols. The greatest relative differences were observed in fracture gap size and plate thickness, with relative errors exceeding 60% for CBCT. Screw length and diameter were measured with higher precision in UHR PCD-CT (mean relative differences < 10%) and yielded superior reproducibility compared to CBCT (κ values for screw diameter as low as 0.46). Overall, UHR PCD-CT outperformed CBCT across most metrics, especially in low-dose settings, supporting its robustness for quantitative postoperative evaluation (Table 2).

**Table 2:** Quantitative assessment of absolute and relative differences (in millimeters and percentages) between radiological measurements and ground truth was performed for fracture gap size, plate thickness and length, as well as screw length and diameter. Interobserver reproducibility between observer A (resident) and observer B (attending) was evaluated using weighted kappa statistics and intraclass correlation coefficients (ICC) with corresponding 95% confidence intervals. Analyses were conducted for each imaging modality (photon-counting computed tomography (PCD-CT) and cone-beam computed tomography (CBCT)) using dose-equivalent standard-dose and low-dose protocols

	Imaging protocol	Absolute difference to ground truth size (Mean ± SD; in mm)	Relative difference to ground truth size (Mean ± SD, as percentage (%)	Inter-observer agreement weighted kappa (κ) and 95% confidence interval (CI)	Inter-observer agreement Intraclass Correlation Coefficient (ICC) and 95% confidence interval (CI)
Fracture gap size	SD-CBCT LD-CBCT UHR-SD-PCD-CT UHR-LD-PCD-CT	1.0 ± 0.01 1.0 ± 0.01	64.6 ± 61.99 70.3 ± 62.45	0.73 (0.59–0.87); *p* < 0.001 0.66 (0.5–0.81); *p* < 0.001	0.91 (0.84–0.95); *p* < 0.001 0.92 (0.86–0.96); *p* < 0.001
		1.0 ± 0.01 1.0 ± 0.01	62.1 ± 59.1 66.3 ± 56.2	0.78 (0.63–0.93); *p* < 0.001 0.84 (0.74–0.95); *p* < 0.001	0.98 (0.97–0.99); *p* < 0.001 0.98 (0.97–0.99); *p* < 0.001
Plate thickness	SD-CBCT LD-CBCT UHR-SD-PCD-CT UHR-LD-PCD-CT	1.8 ± 0.85 1.8 ± 0.83	38.4 ± 46.79 41.2 ± 54.52	0.53 (0.32–0.75); *p* < 0.001 0.50 (0.28–0.72); *p* < 0.001	0.96 (0.93–0.98); *p* < 0.001 0.95 (0.92–0.97); *p* < 0.001
		1.4 ± 0.84 1.4 ± 0.84	38.5 ± 46.67 33.1 ± 45.5	0.79 (0.66–0.93); *p* < 0.001 0.8 (0.65–0.94); *p* < 0.001	0.95 (0.91–0.97); *p* < 0.001 0.97 (0.96–0.99); *p* < 0.001
Plate length	SD-CBCT LD-CBCT UHR-SD-PCD-CT UHR-LD-PCD-CT	0.9 ± 0.79 0.8 ± 0.71	3.8 ± 3.3 3.1 ± 2.47	0.62 (0.36–0.87); *p* < 0.001 0.64 (0.43–0.84); *p* < 0.001	0.99 (0.99-1.0); *p* < 0.001 0.99 (0.99-1.0); *p* < 0.001
		0.7 ± 0.59 0.6 ± 0.57	3.2 ± 2.58 2.6 ± 1.96	0.74 (0.57–0.89); *p* < 0.001 0.81 (0.64–0.98); *p* < 0.001	0.99 (0.99-1.0); *p* < 0.001 0.99 (0.99-1.0); *p* < 0.001
Screw length	SD-CBCT LD-CBCT UHR-SD-PCD-CT UHR-LD-PCD-CT	0.3 ± 0.21 0.4 ± 0.42	4.7 ± 3.47 5.9 ± 6.01	0.79 (0.68–0.91); *p* < 0.001 0.74 (0.61–0.87); *p* < 0.001	0.98 (0.97–0.99); *p* < 0.001 0.98 (0.97–0.99); *p* < 0.001
		0.2 ± 0.11 0.3 ± 0.31	2.8 ± 1.8 4.7 ± 5.09	0.81 (0.69–0.93); *p* < 0.001 0.83 (0.74–0.93); *p* < 0.001	0.98 (0.97–0.99); *p* < 0.001 0.98 (0.97–0.99); *p* < 0.001
Screw diameter	SD-CBCT LD-CBCT UHR-SD-PCD-CT UHR-LD-PCD-CT	0.2 ± 0.14 0.1 ± 0.01	9.8 ± 8.05 6.7 ± 5.23	0.52 (0.32–0.72); *p* < 0.001 0.46 (0.21–0.70); *p* < 0.001	0.99 (0.97–0.99); *p* < 0.001 0.99 (0.98-1.0); *p* < 0.001
		0.1 ± 0.12 0.2 ± 0.16	6.9 ± 4.82 8.7 ± 9.77	0.74 (0.6–0.89); *p* < 0.001 0.75 (0.57–0.92); *p* < 0.001	0.99 (0.98-1.0); *p* < 0.001 0.99 (0.98-1.0); *p* < 0.001

*CBCT* Cone-Beam Computed Tomography, *PCD-CT* Photon-Counting Detector Computed Tomography, *UHR* Ultra-High Resolution, *SD* Standard-Dose, *LD* Low-Dose

## Discussion

This ex vivo comparative study evaluated postoperative fracture assessment in dentomaxillofacial trauma surgery in the presence of titanium-based and bioresorbable osteosynthesis materials, as assessed by UHR PCD-CT and CBCT at different radiation dose levels. UHR PCD-CT images surpassed the CBCT images in both qualitative and quantitative assessments, particularly using low-dose protocols.

Emerging evidence suggests that PCD-CT may be a transformative technology, offering superior diagnostic performance compared to established reference standards in dentomaxillofacial radiology [15–17]. It offers enhanced anatomical visualization with improved contrast-to-noise ratios [11], leading to more accurate detection of pathologies such as mandibular osseous lesions [9]. PCD-CT also significantly reduces implant-related metallic artifacts, which are a concern in postoperative evaluations for oral and maxillofacial surgery [10, 18]. Furthermore, it improves diagnostic accuracy in surgical implant planning at equal or lower radiation doses [10] and shows promising potential in visualizing challenging endodontic tasks [19].

To the best of our knowledge, this is the first study to assess the potential of UHR PCD-CT in the context of oral and maxillofacial trauma surgery. UHR PCD-CT provided consistently excellent visualization of fracture reduction across both standard- and low-dose protocols, highlighting its diagnostic robustness and further supporting its efficiency at reduced radiation doses [15, 17, 20]. The second major finding is the consistently high-quality visualization of osteosynthesis plates and screws using UHR PCD-CT across all radiation dose levels. The sharp delineation of plate edges, screw heads, drive recesses, and threads, with minimal to no metal artifacts at the bone-metal interface supports previous findings demonstrating the artifact reduction effectiveness of this technology [21, 22]. In contrast, CBCT, although still effective at standard dose, demonstrated moderate to poor visualization under low-dose settings, with a notable decline in image sharpness and diagnostic interpretability, supporting the current evidence in comparative studies [9, 10]. Regarding artifact analysis, it should be noted that although only two screws were used for plate fixation in our study, placing four screws to occupy all screw holes of the plate could potentially increase beam-hardening artifacts. To ensure comparability of results, however, the number and placement of screws were kept consistent across all specimens. The superior performance of UHR PCD-CT images compared with CBCT images, particularly when applying low-dose protocols, is of great importance given the increasing emphasis on personalized dose optimization. This aligns with the As Low As Diagnostically Achievable, being Indication-oriented and Patient-specific (ALADAIP) principle, especially for younger or vulnerable patients who require repeated imaging [23].

Bioresorbable plates serve as an alternative to titanium-based osteosynthesis, especially in pediatric patients, medically complex cases, or in anatomically sensitive regions where secondary removal is undesirable [24]. Their radiolucent nature limits direct radiographic depiction on conventional imaging, making assessment of implant integrity and detection of complications more challenging [25]. In our study, bioresorbable plates were not directly visible in any modality; however, PCD-CT’s high spatial resolution and enhanced hard- and soft-tissue contrast allowed indirect assessment via screw drill holes. While plate integrity cannot be fully assessed, this novel diagnostic approach enables clinically relevant evaluation of fracture reduction and supports effective postoperative monitoring and long-term patient management.

Regarding quantitative assessments, UHR PCD-CT demonstrated a clear advantage, with higher accuracy in measuring fracture gap size, plate dimensions, and screw geometry compared with CBCT, particularly in low-dose protocols. The results obtained in this study further support existing evidence of the quantitative reliability of PCD-CT in low-dose imaging protocols [9]. This is likely due to PCD-CT UHR mode’s small pixel size facilitating a high contrast-to-noise ratio, which may serve for dose reduction while maintaining image quality [26]. Notably, CBCT exhibited larger deviations from ground-truth values for thin osteosynthesis materials, with relative errors exceeding 60% for key parameters at low doses. These discrepancies further highlight the limitations of CBCT for precise postoperative evaluation and underscore the potential clinical value of low-dose PCD-CT in clinical cases requiring high measurement accuracy and reliability.

Although this study was conducted ex vivo, in vivo imaging involves additional factors, such as motion artifacts, vascularization, and variable soft tissue composition, that may affect image quality and diagnostic performance, potentially impacting the delineation of osteosynthesis. The lower noise levels and high spatial resolution of PCD-CT help to mitigate these effects [8], while improved visualization of vasculature has been reported [27, 28]. Advances in deep-learning-based artifact-reduction, optimized imaging protocols, faster acquisition times, and enhanced patient preparation are expected to further reduce these limitations. Nevertheless, current in vivo data in dentomaxillofacial radiology remain limited.

The findings of this study highlight PCD-CT as a feasible, high-resolution modality for postoperative assessment in oral and maxillofacial trauma surgery, providing superior image quality even at low radiation doses. Clinically, PCD-CT enables precise perioperative assessment of fracture reduction, fixation, implant stability, and complications such as screw loosening, migration, or adjacent pathologies that are often obscured by metal-induced artifacts.Accurate postoperative visualization of fixation materials can be critical for patient outcomes, as misplaced or unstable screws can compromise fracture stability, delay bone healing, and increase the risk of infection. By facilitating early detection of such complications, PCD-CT supports timely interventions, improves surgical outcomes, and may ultimately reduce morbidity. Furthermore, given the variability in osteosynthesis systems used worldwide, PCD-CTs’ ability to differentiate materials has greater potential to be useful in complex or unclear surgical situations that require precise planning. With growing scientific evidence and increasing adoption in dentomaxillofacial radiology, these UHR PCD-CT findings have the potential to reshape perioperative imaging protocols and lead to the development of indication-specific guidelines, potentially diverging from conventional CT or CBCT-based approaches in cranio-maxillofacial and oral surgery.

Several study limitations must be mentioned. First, although pig mandibles are recognized as a valid and well-established animal model for dental and orofacial research, in vivo conditions cannot be precisely replicated. Second, despite the induction of a total of 36 fractures across six pig mandibles, the relatively small sample size remains a limitation of this study. Therefore, the findings should be interpreted in the context of a pilot study and require validation in larger clinical cohorts and across a broader range of osteosynthesis materials to confirm their applicability in real-world settings, as well as with additional observers of varying experience levels to confirm generalizability. Third, for pig mandibles exceeding the CBCT field of view, two separate scans were required, whereas PCD-CT consistently captured the entire specimen in a single scan. In these cases, the imaging modality was revealed to the observers; however, all observers were blinded to the dose protocol, and the evaluation order was randomized to minimize bias. Future studies designed to fully eliminate the possibility of modality recognition may further enhance the reliability of observer assessments.

## Conclusion

UHR PCD-CT demonstrates superior postoperative fracture assessment in maxillofacial trauma surgery in the presence of titanium-based and bioresorbable osteosynthesis materials compared with CBCT, by offering enhanced visualization and high measurement accuracy, particularly at low radiation doses. As a next step, validation in clinical cohorts is necessary to confirm these findings and support their integration into routine perioperative trauma imaging, ultimately enhancing clinical decision-making.

## Data Availability

No datasets were generated or analysed during the current study.
